# Hepatic epithelioid hemangioendothelioma: A great mimicker

**DOI:** 10.1016/j.ijscr.2018.10.013

**Published:** 2018-10-12

**Authors:** Antoni Llueca, Dolors Piquer, Yasmine Maazouzi, Carmen Medina, Katty Delgado, Anna Serra, Javier Escrig

**Affiliations:** a Multidisciplinary Unit for Abdominal Pelvic Oncology Surgery (MUAPOS), University General Hospital of Castellon, Spain; b Dept. of Medicine, University Jaume I (UJI), Castellon de la Plana, Spain

**Keywords:** Epithelioid hemangioendothelioma, Liver, Fibroid, Hepatic lesion, Advanced ovarian cancer, Adnexal mass

## Abstract

**Introduction:**

Epithelioid hemangioendothelioma is a malignant mesenchymal tumor of unknown etiology. They tend to be asymptomatic or with non-specific symptoms. The lesion is usually multiple and variable size.

**Presentation of case:**

We describe a clinical case of a 23-years-old patient diagnosed with a pelvic mass, a possible uterine fibroid or adnexal mass, and multiple liver lesions that seemed an advanced ovarian cancer presentation and after liver biopsy turned out to be a hepatic epithelioid hemangioendothelioma.

**Discussion:**

It may be confused with a metastatic process in diagnostic imaging. There have been described some possible risk factors but the etiology remains unknown. The prognosis is usually lethal in 50% of cases. The surgical removal of the lesion and liver transplant appear to be the only hope for these patients.

**Conclusion:**

Epithelioid hemangioendothelioma must be part of our differential diagnosis when we find a liver tumour, especially in young women. Treatment is excision of the tumour in limited disease. In the case of unresectable disease are candidates for liver transplantation.

## Highlights

• Epithelioid hemangioendothelioma (EH) is a rare tumour of derived from endothelial cells.
• The initial presentations can be confused with an advanced ovarian cancer.
• Only Histological exam confirms the definitive diagnosis.
• Most patients are candidates for liver transplantation.

## 1 Introduction

Epithelioid hemangioendothelioma (EH) is a rare tumour of low grade of malignancy derived from endothelial cells, involving different organs and soft tissues. Its incidence is less than 1 case per million people per year. The age of presentation is very variable, from 12 to 93 years [1]. The most frequent topographies are the liver and lungs, and predominantly affects the female gender. Its clinical course and prognosis are unpredictable, although its behaviour may be placed between the hemangioma and angiosarcoma. At the time of diagnosis, the disease is usually at an advanced stage. Imaging techniques are essential for the diagnosis, even though they do not allow an exact diagnosis as it can be confused with benign lesions or liver metastases [2]. Histological exam will give us the definitive diagnosis [3]. This work has been done in line with the SCARE criteria [4].

## 2 Case report

A 23-year-old woman, asymptomatic, presented in a gynaecological examination a pelvic tumour in the left iliac fosse. Physical examination showed a 9 cm mobile mass in the left iliac fosse compatible with fibroid or adnexal mass. She was a non-alcoholic, non-smoker with no co-morbid medical or surgical ailment.

Transvaginal ultrasound shows an anteversion uterus with a proliferative endometrium, normal ovaries and a mass of about 9 cm, which does not seem to have a clear relationship with the uterus.

Pelvic magnetic resonance imaging describes a 9 cm semi-solid mass compatible with pedunculated fibroid or primary retroperitoneal lesion. Cerebral MRI shows no abnormalities.

**Fig. 1:**
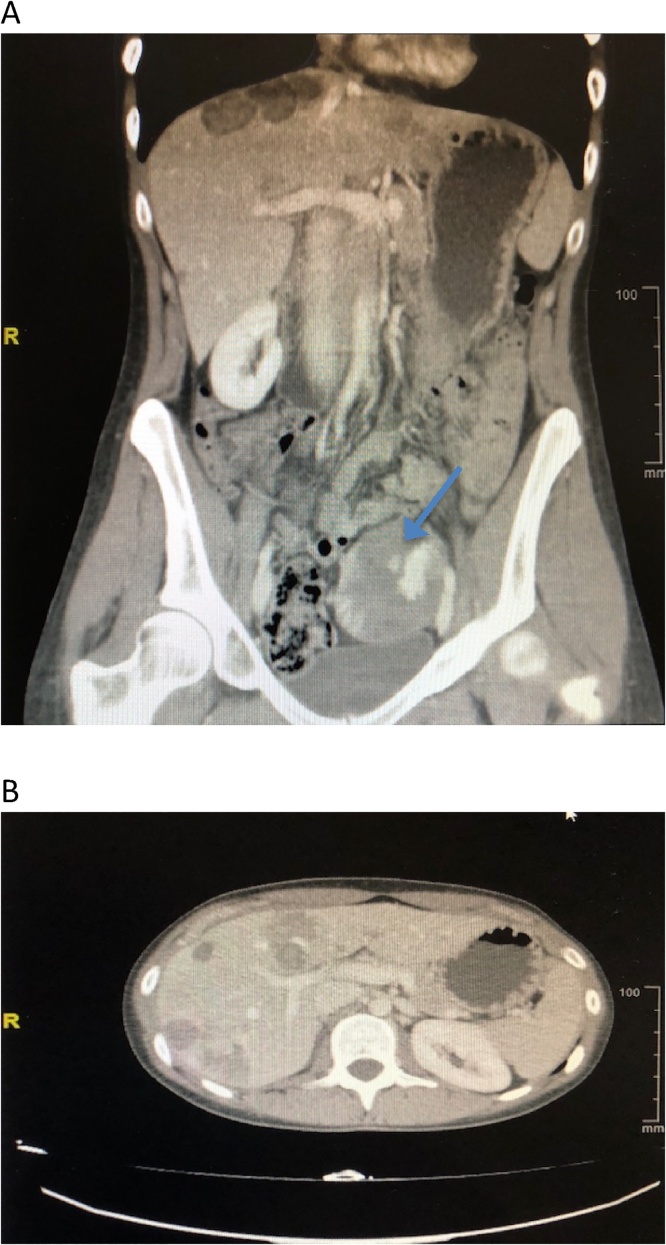
Abdominal CT scan during the portal phase. (A) Pelvic mass (blue arrow). (B) Multiple hepatic lesions.

Abdominal CT scan during the portal phase showed a pelvic mass of 6.5 x 5.5 cm, which seemed to originate from the left ovary, predominantly cystic with solid areas compatible with cystic ovarian neoplasm. Retroperitoneal or pelvic lymph nodes were not identified. There was no ascites. There were multiple bilocular masses informed as metastatic lesions in liver parenchyma. The rest was normal (Fig. 1 A and B).

CEA, Ca 12.5 and Ca 19.9 tumour markers were normal. Alpha-fetoprotein, testosterone, SDHEA and delta-4-androstenedione were also normal.

After discovering these surprising findings, our gynaecologic Tumours Committee decided to perform a guided needle core biopsy of the liver lesions to discart an advanced ovarian cancer. Histologically these lesions corresponded to a vascular tumour compatible with EH.

**Fig. 2:**
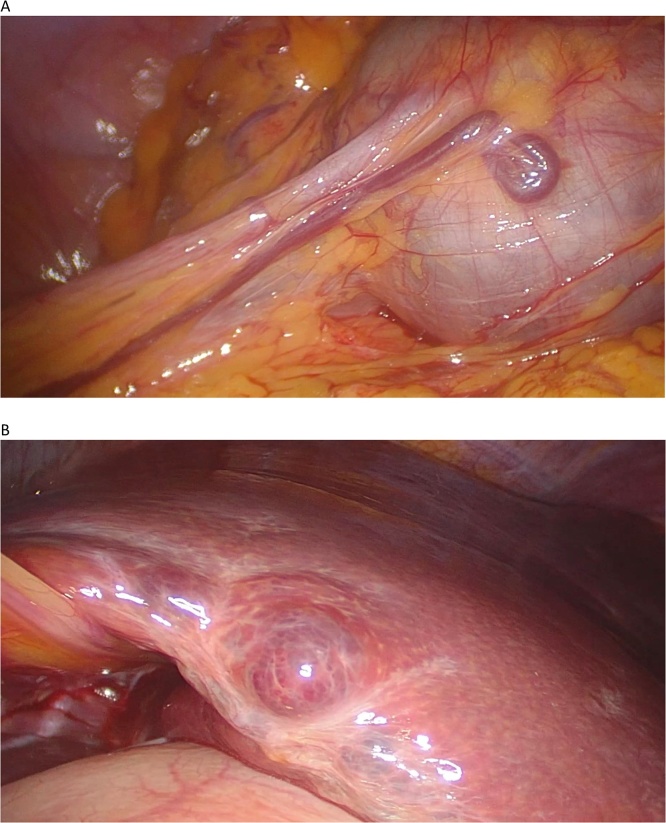
(A) Omental endovascular tumor. (B) Hepatic intraparenchymal lesions.

We decided to perform a diagnostic laparoscopy. During the exploration of the abdominal cavity, an enlarged liver with multiple intra-parenchymal hepatic lesions and a tumour with a large blood vessel originating from the greater omentum could be seen. Partial omentectomy resection, including the mass, was performed and was extracted in an endobag (Fig. 2 A and B).

**Fig. 3:**
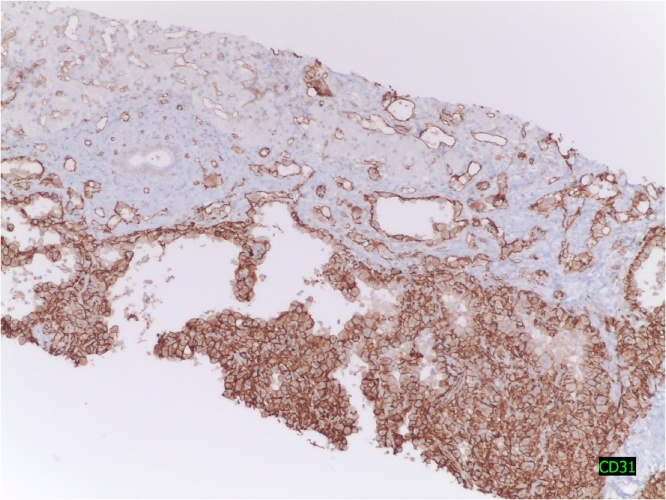
Marked endothelium with CD-31, inmunohistochemical to confirm the diagnosis.

**Fig. 4:**
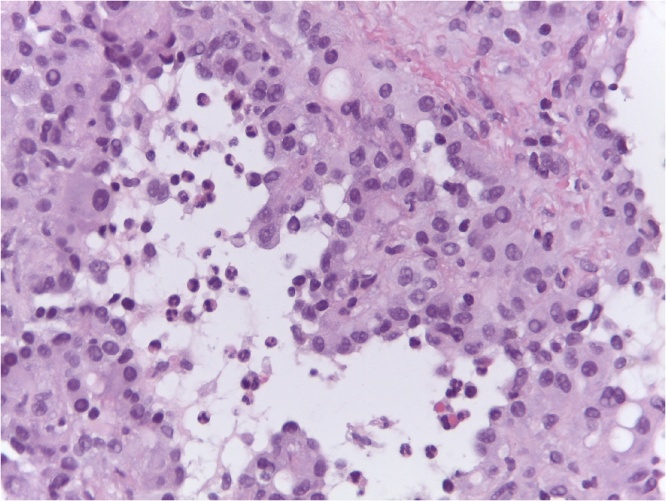
Hematoxylin eosin. The sinusoids are replaced by vascular spaces.

Macroscopically the lump was fleshy, filled with blood and presented whitish areas. Anatomopathological study confirmed an EH. Immunohistochemical profile was CD31+, CD34+, CKAE1/AE3-, CK8/18-, C-kit-, EMA-, vimentin +, progesterone (+ weak, focal), estrogen-, mitotic activity <1 × 10 fields of large increased (Fig. 3, Fig. 4).

After the results, liver resection was discarded due to multicentre lesions, involving pretty much the totality of the liver; making the patient a candidate for liver transplant. Six months later the patient was subjected to a successful liver transplant.

Consent was obtained for the publication of the case report and the images from the patient.

## 3 Discussion

EH is a rare tumour that affects middle-aged women in a ratio of 3:2 with respect to men [3,5,6]. The symptoms are non-existent in the majority of cases, and in some cases, unspecified pain in right upper quadrant, hepatomegaly, and weight loss [7].

There have been possible causal agents or risk factors which have been hypothesized, such as vinyl chloride, polyurethane/silica, oral contraceptives, primary biliary cirrhosis, viral hepatitis, asbestos and alcohol use, among others. However, based on the evidence, the etiology remains unknown [3,8].

In terms of the complementary examinations, more than 84% of the patients present altered liver function, a slightly elevated alpha-fetoprotein and a carcinoembryonic antigen (CEA), which tends to be negative [9]. Ultrasound scan shows hypoecogenic lesions, though they may have a mixt pattern or may even be hyperecogenic [10]. The CT shows hypodense lesions that can retract Glisson’s capsule.

Histopathological diagnosis with routine techniques is difficult, considering differential diagnosis of sclerosing hepatic tumours (hepatocellular carcinoma and cholangiocarcinoma), metastases of carcinomas, particularly those of signet ring cells and angiosarcoma [10].

Histologically characterized for having variable of epithelioid cells and dendritic proportions in the bosom of a stroma with an infiltrate inflammatory joint (85%), and infiltrative nature, which often respects the liver acinar architecture, that is often calcified and sclerosed, and which is particularly prominent in the acinar area [1]. Immunohistochemical techniques demonstrate the endothelial differentiation (positivity of factor VIII in 99% of cases, of CD34 in 94%, of CD31 in 86%, cytoqueratins rarely, and wealth of type IV collagen and laminin) [11,12].

The peculiar natural history of hepatic EH makes difficult the assessment of the effectiveness of the treatments. 50% of patients without treatments survive more than 5 years and the existence of metastasis does not influence the prolongation or shortening of the survival rate. In patients with single or few lesions, surgical excision is the treatment of choice. Patients with extensive, or multiple injuries which are the majority, must be transplanted and the existence of metastasis is not a contraindication [12]. Chemotherapy and radiotherapy have not influenced significantly the course of the disease [13]. Although very useful, arterial embolization in the treatment of hepatocarcinoma has not been able to be evaluated yet [10].

## 4 Conclusion

Therefore, the EH must be part of our differential diagnosis when we find a liver tumour, especially in young women. Treatment is excision of the tumour in limited disease. In the case of unresectable disease are candidates for liver transplantation. A multidisciplinary team and a good management of the Tumours Committee is the most important thing to handle this type of complex cases.

## Conflicts of interest

The authors declare no conflict of interest.

## Source of funding

This work received financial support from d Medtronic University Chair for Training and Surgical Research (University Jaume I - UJI. Castellon, Spain).

## Ethical approval

All procedures performed in studies involving human participants were in accordance with the ethical standards of the local ethics and research committee and followed the Declaration of Helsinki guidelines. Written informed consent was required for collecting data.

Approval by the Ethics and research committee of our institution (CEIm) was obtained. Ref. 012/2017.

## Consent

Written informed consent was obtained from the patient for publication of this case report and accompanying images. A copy of the written consent is available for review by the Editor-in-Chief of this journal on request.

## Author contribution

Study concepts: Antoni Llueca, Dolors Piquer.

Study design:Antoni Llueca.

Data acquisition: Anna Serra, Katty Delgado.

Quality control of data and algorithms: Yasmin Maazouzi.

Data analysis and interpretation: Yasmin Maazouzi.

Manuscript preparation: Antoni Llueca, MC Medina.

Manuscript editing: Antoni Llueca, Dolors Piquer.

Manuscript review: Anna Serra, Javier Escrig.

## Registration of research studies

This is not a first-in-man case report and thus cannot be registered.

## Guarantor

Dr. Llueca is the guarantor of the paper.

## Provenance and peer review

Not commissioned, externally peer reviewed.
